# Will Jordan be closer to UHC after the COVID-19 pandemic?

**DOI:** 10.7189/jogh.10.020360

**Published:** 2020-12

**Authors:** Reem Al-Ajlouni, Abeer Al Rabayah

**Affiliations:** 1 Jordan Breast Cancer Program, Amman, Jordan; 2 King Hussein Cancer Center, Amman, Jordan

Sustainable development requires having healthy people who can drive and maintain development at all levels. However, health, politics, and economics are three interrelated dimensions that need to be balanced. The COVID-19 pandemic showed the importance of maintaining a healthy population to drive the economy within a political context that identifies health as a human right. Therefore, accelerating the move towards Universal Health Coverage (UHC) is central to sustainable development.

Reich et al. recommended in 2016 that countries aspiring to reach UHC should not keep waiting for the perfect moment to move forward [1]. Countries must take advantage of political opportunities, such as those provided by crises, to advance UHC and develop a strategy to manage interest group pressure. As Jordan is committed to UHC, would the COVID-19 pandemic be a new challenge added to the path towards UHC? Or would it be an opportunity that Jordan can build on to strengthen its health care system and become closer to UHC?

This viewpoint aims to describe the opportunities within Jordanian health care system’s response to the COVID-19 pandemic during the period: March-May, 2020, through reflecting on the five core dimensions of health care system performance: Equity, Quality, Responsiveness, Efficiency, and Resilience.

## THE PATH TOWARDS UHC UNDER CRISIS

Experiences from around the globe, showed that significant social, economic, or political changes accelerate UHC. . For example, significant reforms were introduced in Europe by the end of World War II, and one of them was the establishment of the UK NHS [2]. In Indonesia, Turkey, and Thailand, UHC became a national priority following a period of a financial crisis [1]. In Brazil, UHC becames a priority at the time of re-democratization [1]. In the above experiences, periods of the turmoil created opportunities to break through resistance to reforms of some interest groups and allowed innovative approaches to be adopted. According to Reich et al. [1], this has generated broad-based social movements and opportunities for political leaders to mobilize support from diverse groups and create a sense of national solidarity to promote major reforms.

UHC is a complex process thathas many possible pathways, but it is also feasible and achievable regardless of the country’s income level. Moving towards UHC is a long-term policy process that needs both the technical and political knowledge and stakeholders buy-in. The development community is increasingly recognizing that effective technical solutions need sound policymaking, especially for reforms of social and economic nature like UHC.

There have been some positive outcomes from the COVID-19 response, the World-Bank living paper on Social Protection and Jobs Responses to COVID-19 documented real-time actions in key areas of social protection response. It showed that there is a 10-fold increase in social protection measures adopted globally since March 20, 2020. There has been a remarkable uptick in social insurance measures. Financial support to the elderly and persons with disabilities may take the form of additional benefits to those affected by the pandemic or a generalized increase in pensions [3].

## JORDAN RESPONSE TO COVID-19 PANDEMIC: AN OPPORTUNITY FOR UHC

Jordan is an upper-middle-income-country [4]. The Jordanian health care system is considered complex and fragmented. Nevertheless, it has one of the best health care systems in the region due to the stable situation of the country, and due to prioritizing health care in the development plans and projects. Population growth, the epidemiologic shift toward non-communicable diseases, lifestyle changes and increased health care costs are considered significant challenges for the Jordanian health care system.

Jordan’s total health expenditures are considered high. It is equivalent to 8.9% of the GDP. Despite that, only 55% of Jordan’s population is covered by health insurance, both public and private insurance [5,6].

Achieving UHC requires building strong health care systems. The COVID-19 pandemic tested the strength of health care systems around the world. Response experiences might generate some opportunities for strengthening health care systems. We will describe the opportunities within the Jordanian health care system’s response to the COVID-19 pandemic through reflecting on the five core dimensions of health care system performance.

**Equity**: during the COVID-19 public-health emergency, the government provided access to health care services to all Jordanian citizens, all expatriates who live in Jordan and refugees. Suspected and confirmed cases were referred to assigned hospitals for treatment regardless of nationality, age, or gender. Jordanian Expatriates had the option to return home. During the first return phase, the government covered 14 days of quarantine to returned people at different hotels in Amman and the Dead Sea. The ministry of health provided quarantined people with the required health care services. Confirmed cases were transferred to hospitals to get the necessary treatment. In the second phase, the government provided returned Jordanians with residence for quarantine purposes at a reduced cost [7-10]. This experience reflects that health care is a human right by principle in Jordan. Building on this principle is essential to guide future efforts towards UHC.

**Quality:** Hospitals, health facilities, and even health professions have issued guidelines and policies that suit the situation after the pandemic, including, for example, the infection control policies. Furthermore, the ministry of health has developed an evidence-based treatment protocol based on the recommendations of the epidemic response executive committee [8]. This response shows the importance of evidence-based decision making and clinical practice guidelines in providing consistent treatment to all patients. Applying consistent practice guidelines also leads to more equity as treatments are provided to patients per standardized protocols This experience can be a starting point for developing national-based clinical practice guidelines for both communicable and non-communicable diseases.

**Responsiveness:** Collaboration and communication of all partners together (Ministry of Health, Royal Medical Services, Universities, and the private health sector) had an apparent effect on progress on the ground particularly in the example of the lockdown of the city of Irbid. Here committees representing all concerned parties were formed, and the result was quick and efficient response particularly for the field epidemiological surveillance cadres. The government held daily TV press-conferences to provide critical information.. The government regularly updates the response plan to meet any new signals from the field and to match the Jordanian epidemiological status [7-10].

**Efficiency:** Optimizing the utilization of available human and technical resources to combat the epidemic speeded up the response of concerned parties [7-10]. In order to improve the efficiency of services delivery, the role of primary health care is essential, and this crisis is an opportunity to expand its role in Jordan, and build on the existing extensive network of primary and comprehensive health centers across the country. This action is expected to reduce the workload in hospitals and provides additional human resources to help in controlling the pandemic in the long term.

**Figure:**
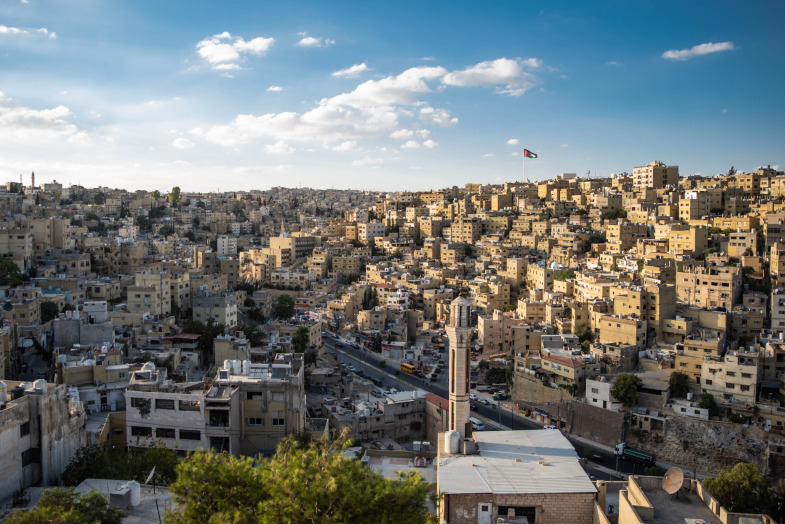
Photo: From Abdulla Ghatasheh, via: https://www.pexels.com/search/jordan.

Also, forming of “Himmat Watan” Fund to mobilize resources to respond to the pandemic repercussions and calling for donations to go directly to Ministry of Health created an appealing way for mobilizing resources for a health cause from both individuals and corporates [7-10].

The use of technology was evident during the crisis. The government created many platforms which speeded up the service-delivery processes. Using user-friendly IT technology solutions is a valuable opportunity for the Jordanian health care system to improve efficiency and future patients’ experiences.

**Resilience:** The government played a role in creating positive interaction with the citizens, touching, and understanding their needs to help them overcome the effects of the crisis and its repercussions. This response had an impact on citizens’ understanding and appreciation of the government’s measures and resulted in active community participation in making these decisions successful. Furthermore, the health care system did not collapse during the COVID-19 response; emergency services and medication delivery to patients with non-communicable diseases continued to be provided [7-10]. Different national initiatives supported the continuity of delivering health care services by the Ministry of Health. This response created a real multi-stakeholder engagement, which represents an opportunity for more collaboration and less fragmentation within our health care system.

## CONCLUSION: WILL JORDAN BE CLOSER TO UHC AFTER THE COVID-19 PANDEMIC?

UHC is not just about achieving cure but also about disease-prevention and health-promotion. There is no better time than ever in contemporary history to emphasize the importance of UHC to public health.

Despite encountered challenges in responding to the COVID-19 pandemic, we think there are some valuable opportunities at the end of the tunnel. We conducted our assessment within the context of the COVID-19 responses that were taken by the Ministry of Health.. We believe that many lessons stemmed from such an experience for Jordan that are considered an eye-opening to improving our health care system to become stronger.

Jordan’s response to the COVID-19 pandemic can induce the needed cultural change for improving our health care system. Moving from bureaucratic culture toward implementing an efficient patient-centered culture can help us in Jordan to have a more robust health care system. It is time for Jordan to prioritize some policy options, including mandatory insurance, creating a mechanism for decreasing fragmentation of resource pooling, investing strategically in human resources for health, developing national treatment guidelines, investment in digitalization, and activating primary health care. Benefting from the lessons learened from the COVID-19 pandemic and implementing those policy options are expected to strengthen our health care system and moves Jordan further steps on the UHC pathway.
